# Supplementary material for: IL-6 inhibition prevents costimulation blockade-resistant allograft rejection in T cell-depleted recipients by promoting intragraft immune regulation in mice
Source: Nat Commun. 2024 Jun 3;15:4309. doi: 10.1038/s41467-024-48574-w (PMC11148062; doi:10.1038/s41467-024-48574-w)
Supplement: Supplementary file 1 — Supplementary information [file 41467_2024_48574_MOESM1_ESM.pdf]

Supplementary Information

IL-6 inhibition prevents costimulation blockade-resistant allograft rejection in T cell-depleted recipients by promoting intragraft immune regulation in mice

Moritz Muckenhuber[1], Konstantinos Mengrelis[1], Anna Marianne Weijler[1], Romy Steiner[1], Verena Kainz[1], Marlena Buresch[1], Heinz Regele[2], Sophia Derdak[3], Anna Kubetz[1] and Thomas Wekerle[1]

[1] Div. of Transplantation, Dept. of General Surgery, Medical University of Vienna, Austria

[2] Clinical Institute of Pathology, Medical University of Vienna, Austria

[3] Core Facilities, Medical University of Vienna, Austria

Supplementary Figure 1:

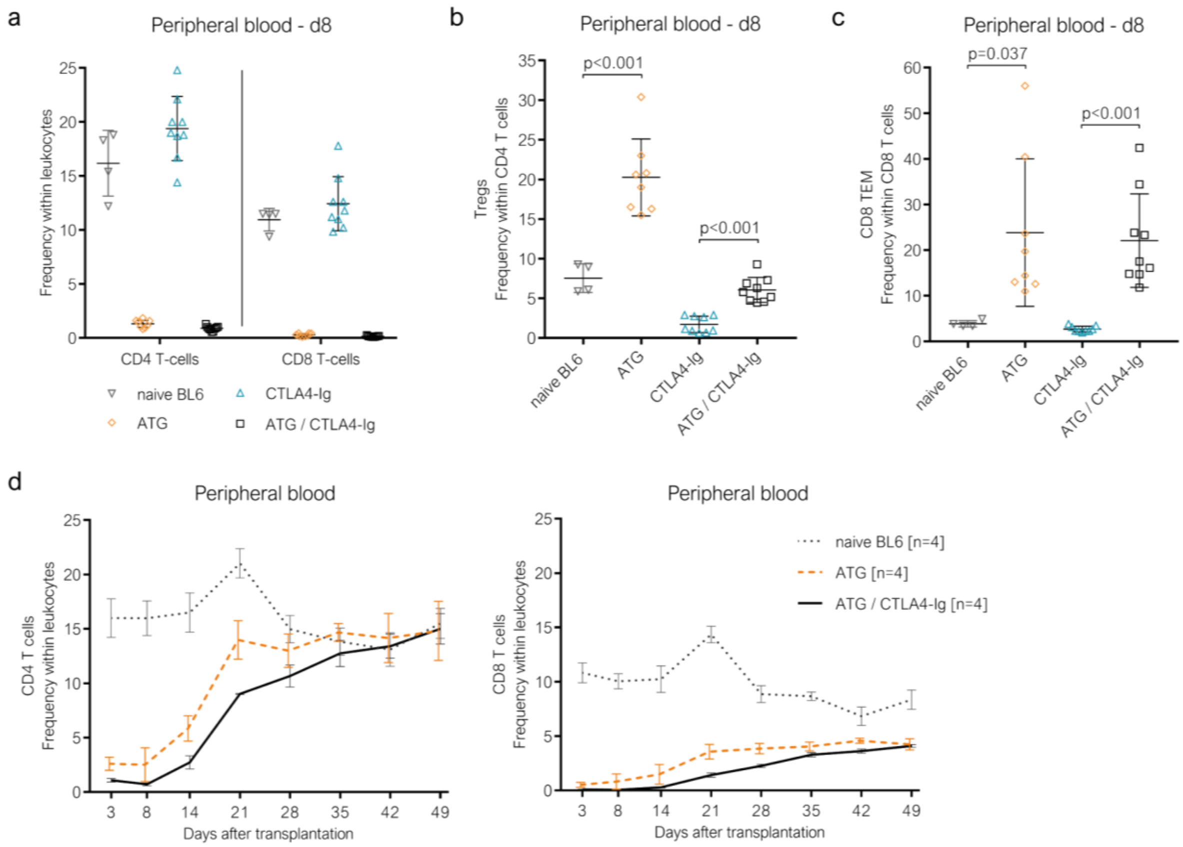

Supplementary Figure 1: *T cell depletion and reconstitution following ATG induction under costimulation blockade*

(a-d) C57BL/6 mice received two doses of anti-thymocyte globulin (ATG, 0.15mg on days 0 and 5) with or without costimulation blockade (CTLA4-Ig). Peripheral blood CD4 and CD8 T cell **(a)**, regulatory T cells (Tregs, $CD4^{+}$ $FOXP3^{+}$) **(b)** and CD8 effector memory T cell (CD8 TEM; $CD8^{+}$ $CD44^{high}$ $CD62L^{low}$) **(c)** frequencies were measured via flow cytometry 8 days after the first ATG injection (naïve BL6 [n=4], ATG [n=8], CTLA4-Ig [n=9], ATG/CTLA4-Ig [n=9]). **(d)** Peripheral blood CD4 and CD8 T cell levels were followed regularly for 7 weeks following ATG administration. Each symbol represents an individual mouse **(a-c)**. Lines indicate group means ± SD. All group comparisons (indicated with brackets) were conducted via unpaired two-sided t-tests.

Supplementary Figure 2:

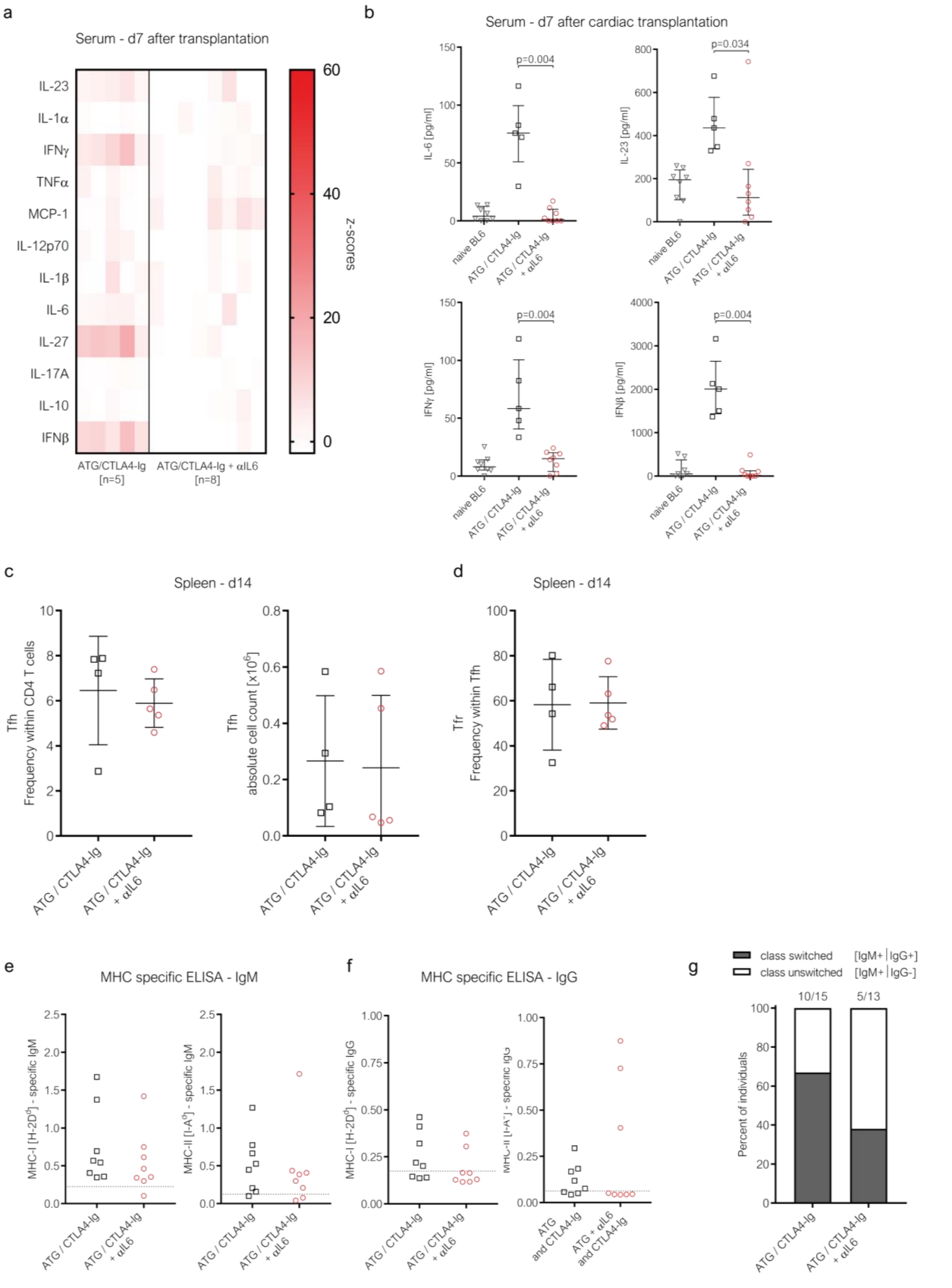

Supplementary Figure 2: *Perioperative IL-6 blockade inhibits pro-inflammatory cytokines and seroconversion of IgM to IgG DSA*

(a-g) C57BL/6 mice received a fully mismatched BALB/c cardiac allograft. **(a and b)** 7 days after transplantation, serum cytokines were assessed in naïve BL6 [n=8] and in recipients treated with ATG/CTLA4-Ig with [n=8] or without [n=5] anti-interleukin-6 (αIL6) using a flow-cytometry based multiplex assay. **(a)** Cytokine levels are depicted descriptively as z-scores. Each column represents an individual mouse. Panel **(b)** illustrates the serum concentrations (pg/ml) of interleukin (IL)-6 (top, left), IL-23 (top, right), interferon-gamma (IFNγ, bottom, left) and interferon-beta (IFNβ, bottom, right), 7 days after cardiac transplantation. **(c and d)** 14 days upon transplantation, T follicular helper cells (Tfh; $CD4^+$ $CD44^+$ $CXCR5^+$ $PD1^+$) **(c)** and T follicular regulatory cells (Tfr; $CD4^+$ $CD44^+$ $CXCR5^+$ $PD1^+$ $FOXP3^+$) **(d)** were quantified in spleens of cardiac allograft recipients using flow cytometry (ATG/CTLA4-Ig [n=4], ATG/CTLA4-Ig + αIL6 [n=5]). Serum from cardiac allograft recipients was isolated at rejection or 100 days after transplantation. **(e-g)** Serum IgM **(e)** and IgG **(f)** antibodies specific for single donor MHC class I (H-$2D^d$) and MHC class II (I-A^d) antigens were quantified via MHC-specific ELISA and are depicted as optical density (OD 405-492nm) (ATG/CTLA4-Ig [n=8], ATG/CTLA4-Ig + αIL6 [n=8]). Dotted lines indicate the limit of detection. Panel **(g)** illustrates the likelihood of seroconversion (from IgM DSA to IgG DSA per group) for recipients within each treatment group. The number of DSA specificities present as IgM and IgG (IgM^+ IgG^+; class switched) was divided by the number of specificities present as IgM but not IgG (IgM^+ IgG^-; class unswitched) for each treatment group (MHC class I and II were pooled). Each symbol represents an individual mouse. Lines indicate group medians ± IQR for non-normal distributed values (panel b) and group means ± SD for normal distributed values (panels c and d). Group comparisons (indicated with brackets) were conducted using a two-sided Mann-Whitney-U test.

Supplementary Figure 3:

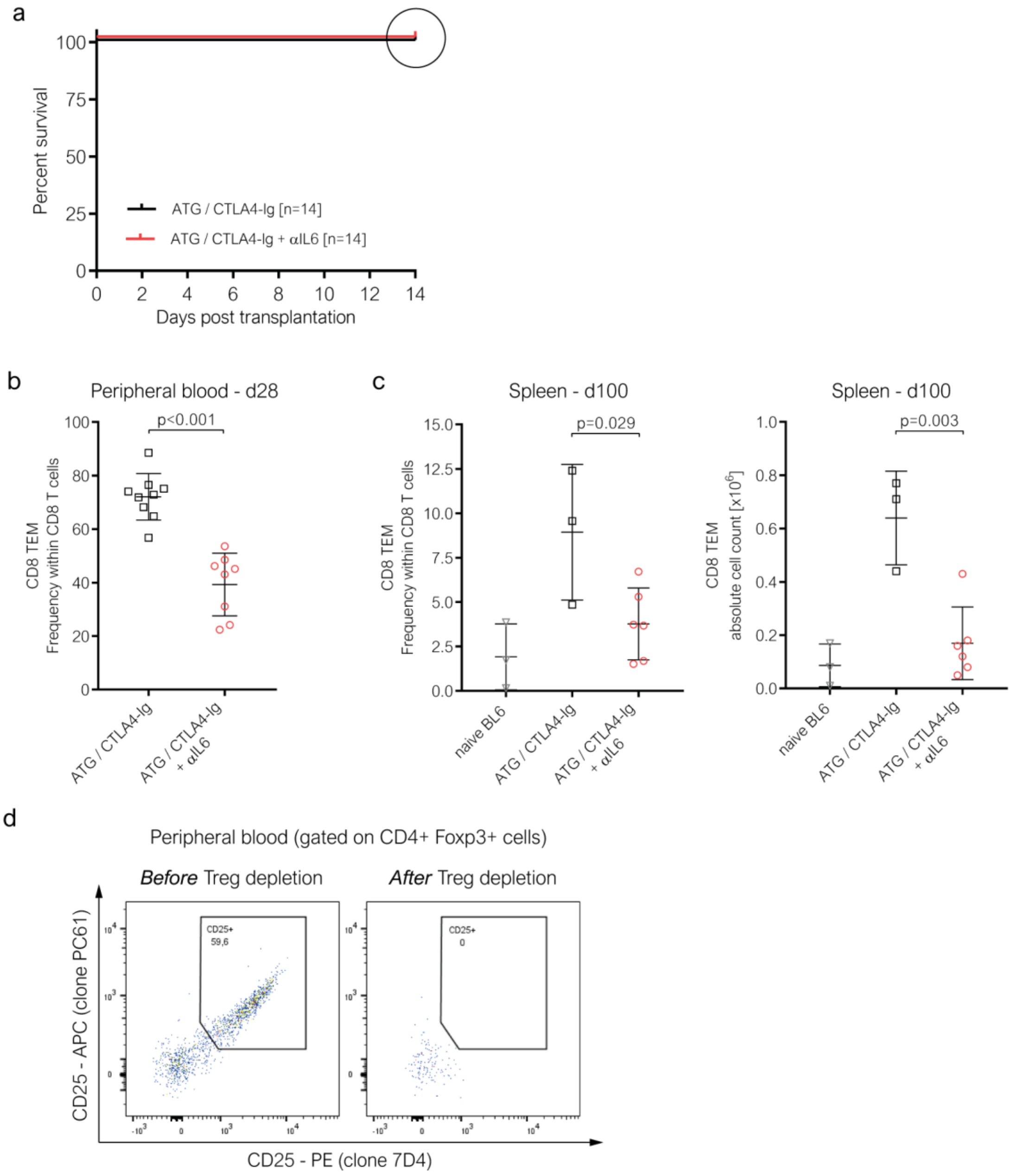

Supplementary Figure 3: *Survival analysis of cardiac allografts explanted for intragraft T cell analysis, memory T cell formation and confirmation of Treg depletion*

(a-d) C57BL/6 mice were grafted with a fully mismatched BALB/c heart under the indicated immunosuppressive regimen. **(a)** Cardiac allografts were explanted 14 days after transplantation (indicated by the circle) for flow cytometric, histological, and transcriptional analysis. Cardiac allograft survival (terminated after 14 days) for each treatment group is depicted as Kaplan-Meier curve. **(b and c)** CD8 effector memory T cells (CD8 TEM; $CD8^{+}$ $CD44^{high}$ $CD62L^{low}$) were quantified in peripheral blood 4 weeks after transplantation (ATG/CTLA4-Ig [n=8], ATG/CTLA4-Ig + αIL6 [n=9]) **(b)** and in the spleen of recipients with long-term surviving allografts 100 days after transplantation (naïve BL6 [n=3], ATG/CTLA4-Ig [n=3], ATG/CTLA4-Ig + αIL6 [n=6]) **(c)**. **(d)** Selected groups of cardiac allograft recipients treated with

ATG/CTLA4-Ig + αIL6 were injected with a depleting anti-CD25 monoclonal antibody (clone: PC61). Regulatory T cell (Treg) depletion was confirmed via flow cytometry of peripheral blood using fluorophore-conjugated PC61 and a second non-competing anti-CD25 monoclonal antibody (clone: 7D4). A representative flow cytometry plot gated on regulatory T cells (Tregs) is shown in panel **(d)**, confirming the absence of CD25-positive Tregs after *in vivo* Treg depletion. Each symbol represents an individual mouse. Lines indicate group means ± SD. All group comparisons (indicated with brackets) were conducted via two-sided unpaired t-test.

Supplementary Figure 4:

a

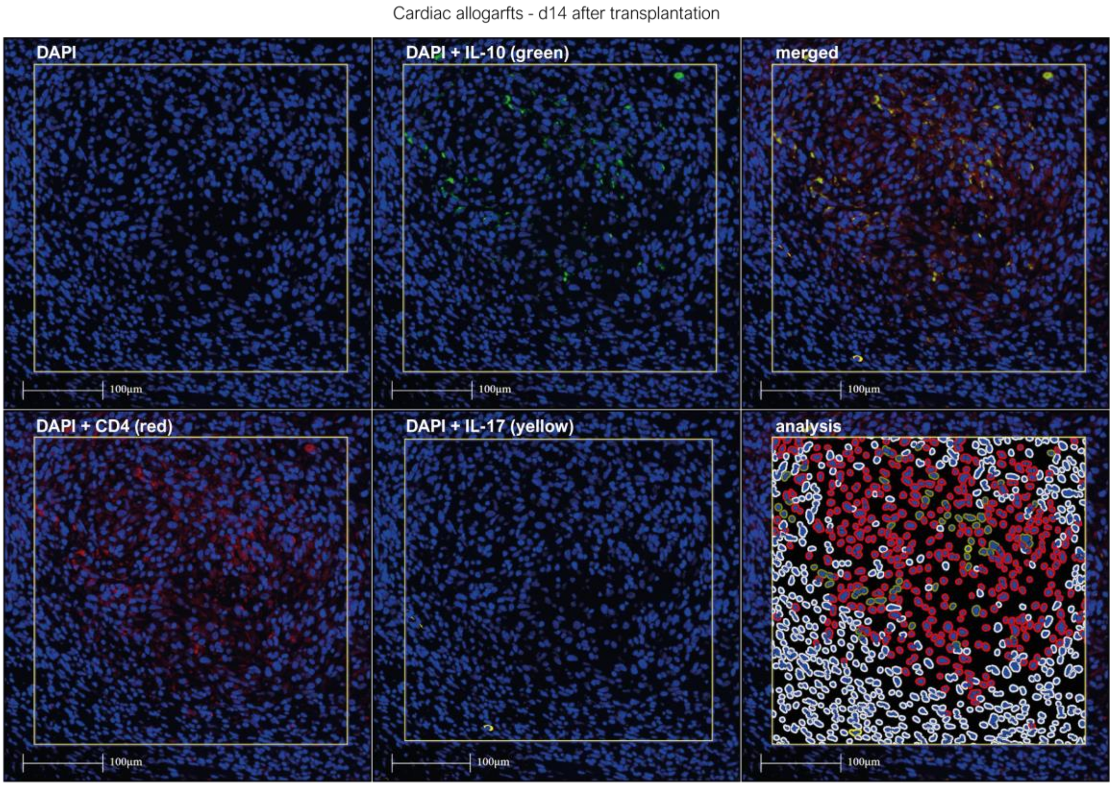

b

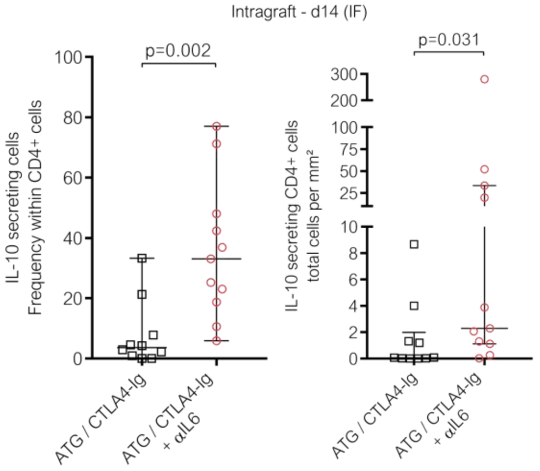

c

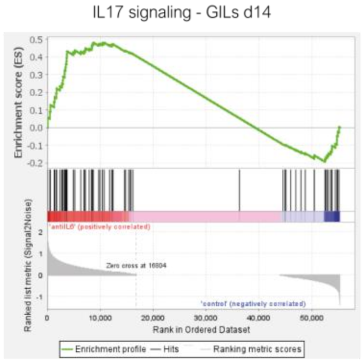

d

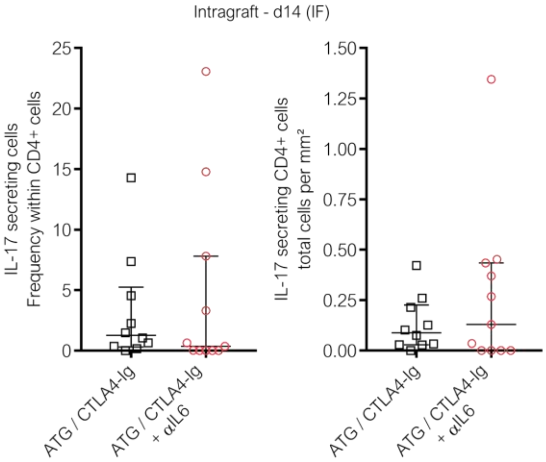

Supplementary Figure 4: *IL-6 blockade increases IL-10 secreting CD4 T cells within cardiac allografts*

(a-d) C57BL/6 mice received a fully mismatched BALB/c cardiac allograft under the indicated immunosuppressive regimens. 14 days after transplantation, the grafts were explanted and graft infiltrating leukocytes (GIL) were analyzed via immunofluorescent microscopy and bulk RNA sequencing. Panel **(a)** shows a representative image of cardiac allografts stained for nuclei (DAPI, blue), CD4 (alexa-fluor 555, red), interleukin-10 (IL-10, alexa-fluor 488, green), and interleukin-17 (IL-17, alexa-fluor 647, yellow) and a representative image of the automated cell-classification during analysis. **(b)** The absolute number (cells per mm^2) and the frequency within $CD4^+$ cells were quantified for IL-10 secreting cells (ATG/CTLA4-Ig [n=10], ATG/CTLA4-Ig + αIL6 [n=11]). **(c)** GIL were isolated from explanted cardiac allografts and flow sorted ($7AAD^-$ $CD45^+$) for bulk RNA sequencing. Genes associated with interleukin-17 (IL17) signaling were compared between ATG/CTLA4-Ig (control, [n=4]) and ATG/CTLA4-Ig + αIL6 (antiIL6, [n=4]) treated recipients via GSEA. **(d)** The absolute number (cells per mm^2) and the frequency within $CD4^+$ cells were quantified for IL-17 secreting cells (ATG/CTLA4-Ig [n=10], ATG/CTLA4-Ig + αIL6 [n=11]). Each symbol represents an individual. Lines indicate the group median ± IQR (as the obtained cell counts and the frequencies did not follow a normal distribution). All group comparisons indicated with brackets were performed using a two-sided Mann-Whitney-U test.

Supplementary Figure 5:

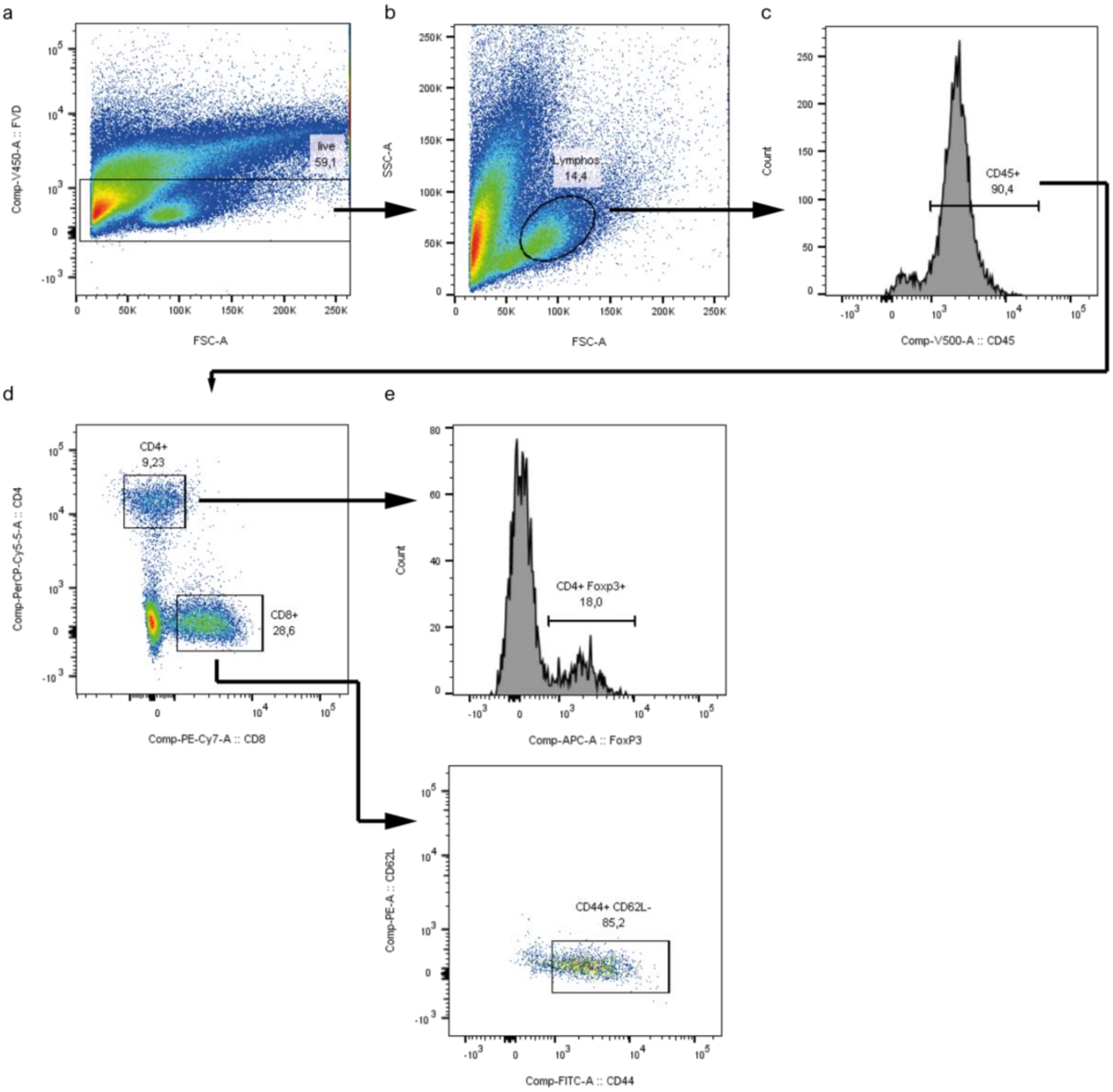

Supplementary Figure 5: Representative *flow cytometry analysis of graft infiltrating leukocytes*

Panels **(a-e)** illustrate the gating strategy for the flow cytometric analysis of graft infiltrating leukocytes isolated from explanted cardiac allografts (corresponding to figures 1c-1d, 3a-3d, 3f-3i in the main text). Upon doublet exclusion, **(a)** fixable viability dye (FVD) negative cells were gated as "live" cells. **(b)** Lymphocytes were gated based on forward (FSC-A) and sideward (SSC-A) scatter, and in **(c)** confirmed to be positive for CD45. **(d)** Thereafter, CD4 and CD8 positive cells were discriminated. Within $CD4^+$ cells, $FOXP3^+$ cells were quantified. Within $CD8^+$ cells, $CD44^{high}$ $CD62L^{low}$ cells were quantified.

Supplementary Figure 6:

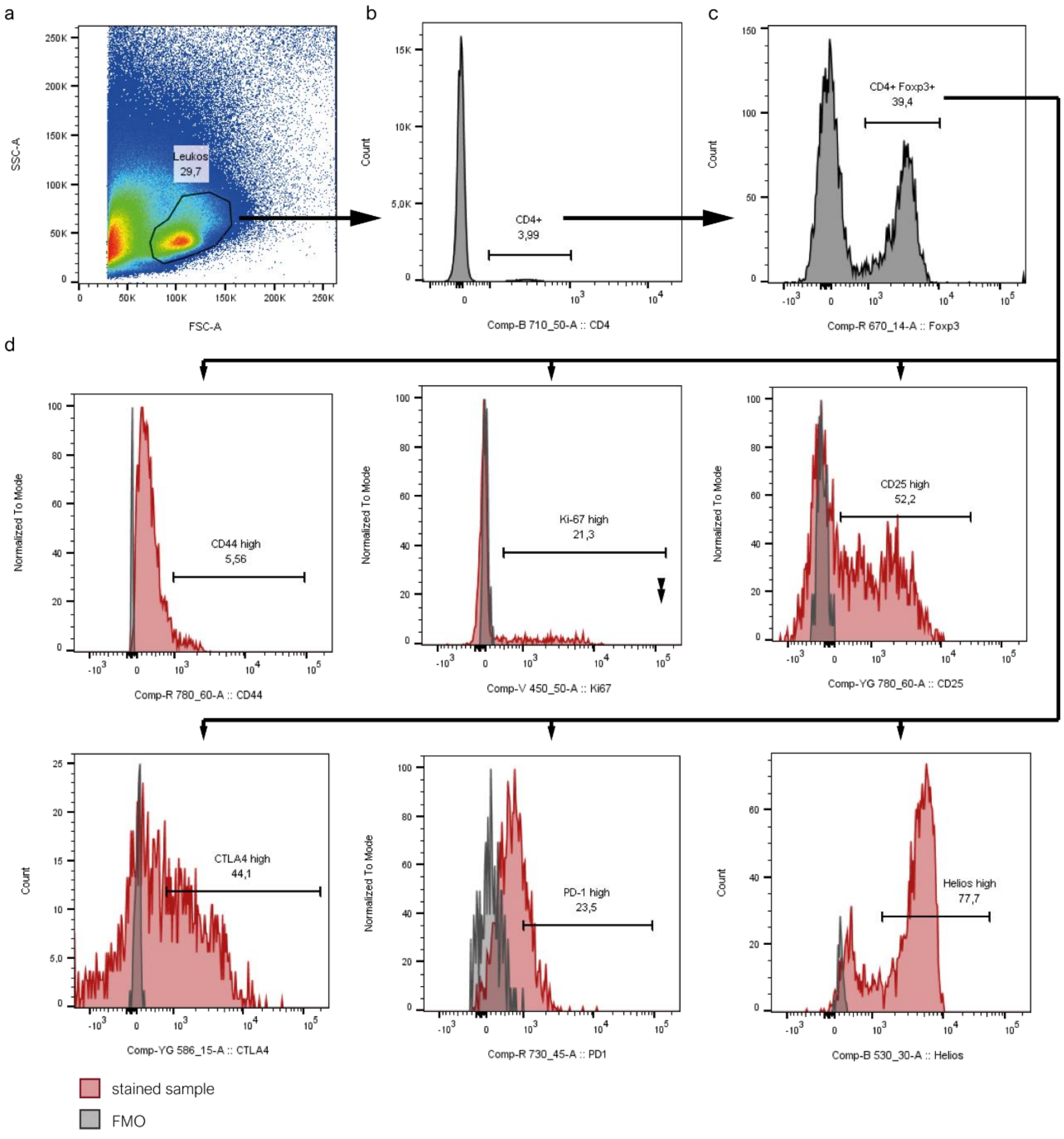

Supplementary Figure 6: Representative *flow cytometric characterization of Tregs*

Panels **(a-d)** illustrate the gating strategy for the flow cytometric analysis of splenic regulatory T cells (Tregs) (corresponding to figures 5c-5h in the main text). **(a)** Leukocytes were gated based on forward (FSC-A) and sideward (SSC-A) scatter. Thereafter $CD4^+$ **(b)** and $CD4^+$ $FOXP3^+$ **(c)** cells were gated (following this gating strategy, Tregs were also quantified within peripheral blood samples corresponding to figures 5a and 5b in the main text). The expression of the functional markers CD44, Ki-67, CD25, CTLA4, PD-1, and Helios was analyzed within $CD4^+$ $FOXP3^+$ cells and is depicted as overlay with control samples lacking the respective staining antibody (i.e. “fluorescence minus one” control samples, FMO) **(d)**.

Supplementary Table 1:

reactivity	method	antigen	clone	conjugate	dilution	supplier	catalogue number
mouse	FC	CD8	53-6.7	FITC	1:20	Biolegend	100706
mouse	FC	CD4	GK1.5	Percp-EFluor710	1:32	Thermo Fisher	14-0041-82
mouse	FC	foxp3	FJK16s	APC	1:16	Thermo Fisher	17-5773-82
mouse	FC	CD62L	MEL-14	PE	1:20	Biolegend	104408
mouse	FC	CD25	PC61	PE-Cy7	1:16	Biolegend	102016
mouse	FC	CD44	IM7	BV510	1:40	BD	560780
mouse	FC	Ki-67	SolA15	BV421	1:16	Thermo Fisher	404-5698-82
mouse	FC	CD80	16-10A1	FITC	1:20	Biolegend	104706
mouse	FC	CD4	RM4-5	APC-Cy7	1:32	Biolegend	100526
mouse	FC	CD11c	N418	PE	1:64	Biolegend	117308
mouse	FC	MHC-II	M5/114.15.2	PE-Cy7	1:128	Biolegend	107630
mouse	FC	CD86	GL1	BV421	1:20	BD	564198
mouse	FC	Helios	22F6	Alexa Fluor 488	1:10	BD	563950
mouse	FC	PD-1	J43	APC-R700	1:10	BD	565815
mouse	FC	CTLA4	UC10-4F10-11	PE	1:10	BD	561718
mouse	FC	CD45.2	104	BV510	1:64	BD	740131
mouse	FC	pan IgG	poly4053	PE	1:20	Biolegend	405307
mouse	FC	ICOS	C398.4A	FITC	1:10	Thermo Fisher	11-9949-82
mouse	FC	PD-1	RMP1-30	PE	1:10	Thermo Fisher	12-9981-82
mouse	FC	CXCR5	L138D7	PE-Cy7	1:8	Biolegend	145516
mouse	IF	CD8	53-6.7	unconjugated	1:200	Biolegend	100702
mouse	IF	foxp3	EPR22102-37	unconjugated	1:200	Abcam	ab215206
mouse	IF	IL-17	polyclonal	unconjugated	1:200	Abcam	ab91649
mouse	IF	IL-10	JES5-2A5	unconjugated	1:200	Abcam	ab189392
mouse	IF	CD4	polyclonal	unconjugated	1:300	R&D	AF554

Supplementary Table 1: *List of antibodies*

Supplementary table 1 lists all antibodies and their respective dilution when used for flow cytometry (FC) or immunofluorescent microscopy (IF) within this manuscript.
