# Supplementary material for: IL-6 inhibition prevents costimulation blockade-resistant allograft rejection in T cell-depleted recipients by promoting intragraft immune regulation in mice
Source: Nat Commun. 2024 Jun 3;15:4309. doi: 10.1038/s41467-024-48574-w (PMC11148062; doi:10.1038/s41467-024-48574-w)
Supplement: Supplementary file 3 — Reporting Summary [file 41467_2024_48574_MOESM3_ESM.pdf]

nature portfolio

Corresponding author(s): Thomas Wekerle
Last updated by author(s): 03.04.2024

Reporting Summary

Nature Portfolio wishes to improve the reproducibility of the work that we publish. This form provides structure for consistency and transparency in reporting. For further information on Nature Portfolio policies, see our Editorial Policies and the Editorial Policy Checklist.

Statistics

For all statistical analyses, confirm that the following items are present in the figure legend, table legend, main text, or Methods section.

n/a	Confirmed	
☐	☒	The exact sample size (*n*) for each experimental group/condition, given as a discrete number and unit of measurement
☐	☒	A statement on whether measurements were taken from distinct samples or whether the same sample was measured repeatedly
☐	☒	The statistical test(s) used AND whether they are one- or two-sided<br>*Only common tests should be described solely by name; describe more complex techniques in the Methods section.*
☒	☐	A description of all covariates tested
☐	☒	A description of any assumptions or corrections, such as tests of normality and adjustment for multiple comparisons
☐	☒	A full description of the statistical parameters including central tendency (e.g. means) or other basic estimates (e.g. regression coefficient) AND variation (e.g. standard deviation) or associated estimates of uncertainty (e.g. confidence intervals)
☐	☒	For null hypothesis testing, the test statistic (e.g. *F*, *t*, *r*) with confidence intervals, effect sizes, degrees of freedom and *P* value noted<br>*Give P values as exact values whenever suitable.*
☒	☐	For Bayesian analysis, information on the choice of priors and Markov chain Monte Carlo settings
☒	☐	For hierarchical and complex designs, identification of the appropriate level for tests and full reporting of outcomes
☒	☐	Estimates of effect sizes (e.g. Cohen's *d*, Pearson's *r*), indicating how they were calculated

Our web collection on statistics for biologists contains articles on many of the points above.

Software and code

Policy information about availability of computer code

Data collection	The flow cytometry data presented in this publication were obtained on a BD LSR Fortessa and a BD FACS Canto using the BD FACS Diva software v9.0 (BD). Flow cytometric sorting was performed on a BD FACSAria fusion. Immunofluorescent microscopy images were obtained using the NIS elements AR software v4.51 (Nikon) and the highplex fl module v4.2 of HALO v3.6.4134.362 (indica labs). ELISA data was obtained on a Victor microplate reader using the Magellan v7.2 software (Tecan). For the quality control of RNA samples prior to RNA sequencing, a Bioanalyzer 2100 (Agilent) with the Agilent 2100 Expert Software version B.02.12 was used. RNA samples were sequenced on a NextSeq500 (Illumina) using the NextSeq System Suite v2.2.0 software (Illumina).
Data analysis	The flow cytometry data was analyzed using FlowJo v10 (BD) with the open-source plugins FlowSOM_v3.0.18 (PMID: 25573116) and UMAP_v3.1 available via bioconductor. The legendplex (Biolegend) cytokine multiplex array was analyzed using the Legendplex data analysis software suite (Qognit). Histological scans of cardiac allograft tissue sections were analyzed using the case center version 2.9 software (3D histech). Upon RNA sequencing, reads were aligned to the mouse reference genome version GRCm38 with Gencode mV23 annotations using STAR aligner version 2.6.1a (PMID: 23104886) in 2-pass mode. Reads per gene were counted by STAR, and differential gene expression was calculated using DESeq2 version 1.22.2 (PMID: 25516281). TPM were generated by RSEM (PMID: 21816040). Statistical analyses were performed using SPSS version 25 (IBM) and GraphPad Prism version 8 (Dotmatics).<br>Graphical illustrations in figures 1a, 2a, and 4a were generated online using Biorender under the publication licenses: YL259RBWA4, RS259RBQHM, UE26GKIC5E

For manuscripts utilizing custom algorithms or software that are central to the research but not yet described in published literature, software must be made available to editors and reviewers. We strongly encourage code deposition in a community repository (e.g. GitHub). See the Nature Portfolio guidelines for submitting code & software for further information.

Data

Policy information about availability of data

All manuscripts must include a data availability statement. This statement should provide the following information, where applicable:

- Accession codes, unique identifiers, or web links for publicly available datasets
- A description of any restrictions on data availability
- For clinical datasets or third party data, please ensure that the statement adheres to our policy

The datasets generated during and/or analyzed during the current study are available from the corresponding author on request. RNA sequencing data that support the findings of this study are deposited in the NCBI GEO/SRA repository (https://www.ncbi.nlm.nih.gov/geo/query/acc.cgi?acc=GSE241472) under the accession number GSE241472 and are publically available. Source data are provided with this paper.

Research involving human participants, their data, or biological material

Policy information about studies with human participants or human data. See also policy information about sex, gender (identity/presentation), and sexual orientation and race, ethnicity and racism.

Reporting on sex and gender	The data described in this manuscript does not include any human participants, their data or their biological material.
Reporting on race, ethnicity, or other socially relevant groupings	see above
Population characteristics	see above
Recruitment	see above
Ethics oversight	no human participants, their data or their biological material were included in the reported study

Note that full information on the approval of the study protocol must also be provided in the manuscript.

Field-specific reporting

Please select the one below that is the best fit for your research. If you are not sure, read the appropriate sections before making your selection.

☒ Life sciences ☐ Behavioural & social sciences ☐ Ecological, evolutionary & environmental sciences

For a reference copy of the document with all sections, see nature.com/documents/nr-reporting-summary-flat.pdf

Life sciences study design

All studies must disclose on these points even when the disclosure is negative.

Sample size	Data from previous experiments (PMID: 27184870) was used to determine the minimal group size required for the graft survival analyses: These data demonstrate a 100-day survival rate of 10% under CTLA4-Ig monotherapy. For this project, we defined the minimal biologically relevant effect as 80% 100-day survival (hazard ratio: 8) for any intervention group (that aims to prevent costimulation-blockade resistant rejection). Consequently, a minimal sample size of 8 animals per group was calculated to be necessary to detect such an effect with 80% power using a log-rank test at a two-sided significance level of 0.05.<br>For mechanistical analyses, 4-6 mice were analyzed per group. Relevant findings were repeated two to three time resulting in sample sizes between 8 and 13 animals per group to compensate for greater variability.
Data exclusions	One mouse in the graft survival analysis of the ATG/CTLA4-Ig group was censored 80 days after transplantation (Fig. 1b and 2b) as it died of unknown cause with a viable cardiac allograft.<br>Two samples were excluded from the analysis of graft infiltrating leukocytes 14 days after transplantation (both in the ATG/CTLA4-Ig group) because of a technical failure during tissue digestion resulting in no cells being isolated from the grafts. Similarly, isolation of graft infiltrating leukocytes failed in one allograft of the ATG/CTLA4-Ig + anti-IL6 group at the end of follow-up.<br>Three samples (two in the ATG/CTLA4-Ig + anti-IL6 group, one in the ATG/CTLA4-Ig group) were excluded from the histological analysis (ISHLT scores and immunofluorescent microscopy) because of a technical failure during paraffin embedding.
Replication	Experiments were typically performed in groups of 4-6 biological replicates (i.e. mice). All relevant in vivo experiments were repeated at least two times and all replications were successful.
Randomization	All mice were randomly allocated to the indicated experimental groups.
Blinding	The investigators were not blinded to the group allocation of the mice due to the complex therapeutic protocol as this, if done in an investigator-blinded approach, would have exceeded our resources.<br>The histological analyses were performed by a pathologist who was blinded to the experimental background of the samples.

Reporting for specific materials, systems and methods

We require information from authors about some types of materials, experimental systems and methods used in many studies. Here, indicate whether each material, system or method listed is relevant to your study. If you are not sure if a list item applies to your research, read the appropriate section before selecting a response.

Materials & experimental systems

n/a	Involved in the study
☐	☒ Antibodies
☒	☐ Eukaryotic cell lines
☒	☐ Palaeontology and archaeology
☐	☒ Animals and other organisms
☒	☐ Clinical data
☒	☐ Dual use research of concern
☒	☐ Plants

Methods

n/a	Involved in the study
☒	☐ ChIP-seq
☐	☒ Flow cytometry
☒	☐ MRI-based neuroimaging

Antibodies

Antibodies used

species reactivity - antigen - clone - fluorophore - catalog number - manufacturer - PRID

mouse - CD8 - 53-6.7 - FITC - 100706 - Biolegend - AB_312745
mouse - CD4 - GK1.5 - Percp-EFluor710 - 14-0041-82 - Thermo Fisher - AB_467063
mouse - foxp3 - FJK16s - APC - 17-5773-82 - Thermo Fisher - AB_469457
mouse - CD62L - MEL-14 - PE - 104408 - Biolegend - AB_313095
mouse - CD25 - PC61.5 - PE-Cy7 - 102016 - Biolegend - AB_312865
mouse - CD44 - IM7 - BV510 - 560780 - BD - AB_1937328
mouse - Ki-67 - SolA15 - BV421 - 404-5698-82 - Thermo Fisher - AB_2925532
mouse - CD80 - 16-10A1 - FITC - 104706 - Biolegend - AB_313127
mouse - CD4 - RM4-5 - APC-Cy7 - 100526 - Biolegend - AB_312727
mouse - CD11c - N418 - PE - 117308 - Biolegend - AB_313777
mouse - MHC-II - M5/114.15.2 - PE-Cy7 - 107630 - Biolegend - AB_2069376
mouse - CD86 - GL1 - BV421 - 564198 - BD - AB_2738663
mouse - Helios - 22F6 - Alexa Fluor 488 - 563950 - BD - AB_2738505
mouse - PD-1 - J43 - APC-R700 - 565815 - BD - AB_2739366
mouse - CTLA4 - UC10-4F10-11 - PE - 561718 - BD - AB_395005
mouse - CD45.2 - 104 - BV510 - 740131 - BD - AB_2739888
mouse - pan-IgG - poly4053 - PE - 405307 - Biolegend - AB_315010
mouse - ICOS - C398.4A - FITC - 11-9949-82 - Thermo Fisher - AB_465458
mouse - PD-1 - RMP1-30 - PE - 12-9981-82 - Thermo Fisher - AB_466290
mouse - CXCR5 - L138D7 - PE-Cy7 - 145516 - Biolegend - AB_2562210
mouse-CD8-53-6.7-unconjugated-100702-Biolegend-AB_312741
mouse-foxp3-EPR22102-37-unconjugated-ab215206-Abcam
mouse-IL-17-polyclonal-unconjugated-ab91649-Abcam
mouse-IL10-JES5-2A5-unconjugated-ab189392-Abcam
mouse-CD4-polyclonal-unconjugated-AF554-R&D

Validation

All antibodies used are commercially available and were validated for use in flow cytometry by the manufacturer. All antibodies were adequately titrated usind murine splenocytes.

Animals and other research organisms

Policy information about studies involving animals; ARRIVE guidelines recommended for reporting animal research, and Sex and Gender in Research

Laboratory animals

Female C57BL/6 (H-2b, strain code: 027) and BALB/c (H-2d, strain code: 028) mice between 12 and 16 weeks of age were purchased from Charles River Laboratories (Germany) and Janvier-Labs (France). All mice were co-housed under specific-pathogen free (SPF) conditions in individually ventilated cages (up to 5 animals per cage) at 21°C room temperature with a 12-hour light-dark cycle at the core facility laboratory animal breeding and husbandry of the Medical University of Vienna. Mice were handled in accordance with national and international guidelines of laboratory animal care and euthanized via cervical dislocation.

Wild animals

The reported study did not involve wild animals

Reporting on sex

The reported study did not include sex-based analyses. Only female mice were included in the experiments as the territorial behaviour of male mice would lead to a significantly higher drop-out rate, particularly after surgery.

Field-collected samples	The reported study did not include samples collected from the field
Ethics oversight	All animal experiments wer approved by the local review board of the Medical University of Vienna and by the Austrian Federal Ministry of Science, Research and Economy under vote number: BMWFW-66.009/0118-WF/V/3b/2016

Note that full information on the approval of the study protocol must also be provided in the manuscript.

Flow Cytometry

Plots

Confirm that:

☒ The axis labels state the marker and fluorochrome used (e.g. CD4-FITC).

☒ The axis scales are clearly visible. Include numbers along axes only for bottom left plot of group (a 'group' is an analysis of identical markers).

☒ All plots are contour plots with outliers or pseudocolor plots.

☒ A numerical value for number of cells or percentage (with statistics) is provided.

Methodology

Sample preparation	Spleen samples were pushed through a 70µm restrainer to create a single cell suspension. Upon centrifugation at 1200RPM for 10 minutes, red blood cell lysis was performed by adding 1ml of red blood cell lysing buffer (Sigma). Lysis was stopped by adding 20ml PBS (Gibco). Cell counts were determined using a CASY cell counter (OLS).<br>Peripheral blood samples were heparinized to prevent clotting and were kept on ice during processing. 200µl of blood were transferred into a 15ml Falcon tube filled with 9ml ddH2O for osmotic lysis of red blood cells. Lysis was stopped after 20 secondy by adding 1ml of 10x Hanks balanced salt sollution (Sigma).<br>For analysis of graft infiltrating leukocytes, explanted cardiac allografts were enzymatically digested using a mouse tumor dissociation kit (Miltenyi) following the manufacturer's instructions. Briefly, grafts were cut into small pieces and incubated for 40min on 37°C with an enzyme mixture. Upon washing with 10ml PBS + 2% BSA, residual red blood cells were lysed (using the same procedure as described for spleen samples) and the number of cells determined using a CASY cell counter (OLS).<br>1x10^6 cells per sample and antibody panel were transferred into a 5ml polystyrene tube (Fisher scientific) and incubated with 100µl of a mastermix containing the indicated flow cytometry antibodies (diluted in PBS + 0.5% BSA) for 30min on 4°C in the dark. For intracellular staining, a foxp3/transcription factor staining buffer set (eBioscience) was used according to the manufacturers instructions. Briefly, after surface staining and washing, 1x10^6 cells were incubated with 1ml of the diluted fix/perm concentrate for 1h on 4°C in the dark. Upon washing with permeabilization buffer, intracellular staining was performed simmilarly to surface staining.
Instrument	BD LSR Fortessa (BD) and BD FACS Canto II (BD) for flow cytometry and BD FACSAria fusion for flow-cytometric cell sorting
Software	BD FACS Diva software v9.0 (BD) was used for data acquisition and FlowJo v10 (BD) was used for analysis
Cell population abundance	For RNA sequencing of sorted graft infiltrating leukocytes, cardiac allografts were digested as described above and stained for flow cytometric sorting. Sorting was performed on a BD FACSAria fusion. Post-sort purity analysis demnostrated >90% of the sorted cells being viable leukocytes (CD45+ 7AAD-).
Gating strategy	In general, all gating strategies started by doublet exclusion using forward scatter (FSC) height (FSC-H) vs. area (FSC-A), followed by dead-cell exclusion (7AAD positive cells or fixable viability dye positive cells in case cells were permeabilized), and gating for lymphocytes using FSC-A (x-axis) vs. sideward scatter area (SSC-A).<br><br>Figure 1 and 3 - Treg/CD8 TEM Quantification in spleen or peripheral blood samples and within graft infiltrating leukocytes:<br>lymphocytes --> CD45+ --> CD4+ cells --> Foxp3+ cells identified as Tregs<br>--> CD8+ cells --> CD44-high CD62L-low cells identified as CD8 TEM cells<br><br>Figure 5 - Phenotypical analysis of splenic Tregs:<br>lymphocytes --> CD4+ cells --> Foxp3+ cells --> CD44-high Tregs<br>--> Ki-67-high Tregs<br>--> CD25-high Tregs<br>--> CTLA4-high Tregs (stained on the surface and intracellularly)<br>--> PD-1-high Tregs<br>--> Helios-high Tregs<br><br>The boundries between "positive" and "negative" populations were based on "fluorescence minus one" (FMO) stainings if the populations were not clearly separated.

☒ Tick this box to confirm that a figure exemplifying the gating strategy is provided in the Supplementary Information.
